# Delayed Access Is a Migraine Risk Factor: Precision Triage as the Missing Link in Modern Headache Care

**DOI:** 10.7759/cureus.110474

**Published:** 2026-06-08

**Authors:** Stjepan Skudar

**Affiliations:** 1 Family Medicine, Zagreb West Health Center, Zagreb, HRV

**Keywords:** access to care, artificial intelligence, calcitonin gene-related peptide, digital health, headache medicine, implementation science, medication overuse, migraine, precision triage, preventive treatment

## Abstract

Migraine care has become scientifically precise while many patient pathways remain operationally imprecise. Diagnostic criteria, global burden data, migraine-specific preventive therapies, and emerging digital tools are available, yet many patients still experience delayed diagnosis, underestimated disability, escalating acute medication use, and late access to prevention. This perspective argues that delayed access should be understood not merely as an administrative barrier, but as a modifiable risk factor that may contribute to migraine progression, medication overuse, and avoidable disability. It proposes precision triage as a practical implementation framework for connecting headache classification, burden assessment, medication-overuse risk, rational preventive treatment selection, timely referral, and accountable use of digital health tools. The central claim is that modern migraine care must shift from disconnected visits to a pathway-based model that asks what each patient needs next and how soon. Such a shift may help translate existing headache knowledge into faster, fairer, and more clinically responsive care.

## Editorial

Introduction

Migraine medicine has become scientifically precise while remaining operationally imprecise. Clinicians share a detailed diagnostic language through the International Classification of Headache Disorders, third edition [1]. The global burden literature consistently demonstrates that migraine is a leading cause of disability, not a benign inconvenience [2,3]. The American Headache Society now supports calcitonin gene-related peptide-targeting therapies as first-line preventive options when clinically appropriate [4]. However, many patients still reach effective care only after years of diagnostic uncertainty, escalating acute medication use, work impairment, emergency visits, and preventable loss of function.

This mismatch suggests a provocative but practical thesis: delayed access is not merely an administrative inconvenience in migraine care; it is a modifiable risk factor. When diagnosis is late, disability is underestimated, prevention is postponed, or acute medication use is allowed to rise without structured follow-up, the health system may unintentionally participate in migraine chronification. Migraine progression should not be framed only as biology unfolding inside the patient. It can also be understood as biology interacting with delayed recognition, delayed prevention, and delayed escalation.

From an applied sciences perspective, the problem is not only what headache medicine knows, but how reliably that knowledge becomes usable inside real clinical systems. In headache care, the current implementation gap is no longer explained only by the lack of treatments. The more urgent problem is pathway failure: patients are classified too late, stratified too crudely, escalated too slowly, and followed too inconsistently. Modern migraine care therefore needs not another abstract promise of personalization, but a practical method for deciding what each patient needs next.

Discussion

I propose precision triage as that missing method. Precision triage is not a biomarker, device, or algorithm. It is a disciplined clinical and organizational approach in which every migraine encounter answers five questions: What is the most likely headache diagnosis, and are there red flags? How much burden does the disease create beyond attack frequency? Is the patient stable, worsening, or at risk of medication overuse or chronification? What intervention is most appropriate now? What follow-up or digital support is needed so that deterioration is not discovered months or years too late?

Migraine severity is often flattened into monthly headache days. This is necessary for classification and treatment monitoring, but inadequate for triage. Two patients with six migraine days per month may live different diseases. One may recover predictably; another may lose wages, avoid light, vomit during work, use acute medication repeatedly, fear the next attack, and become less present at home. If both are triaged by frequency alone, care appears objective while missing the actual burden.

Medication-overuse headache illustrates the failure most clearly. It is often discussed as a complication of excessive acute medication use, but it should also be viewed as a signal of insufficient prevention and weak follow-up [5]. Patients usually do not overuse acute medication because they misunderstand migraine. They often do so because acute medication is the only intervention that has been made available quickly enough to preserve employment, caregiving, education, or basic daily function. Precision triage would treat rising acute medication days as an early warning sign requiring a plan, not a late-stage reason for blame.

The 2024 American Headache Society position statement on calcitonin gene-related peptide-targeting therapies makes delayed escalation harder to justify [4]. This does not mean that every patient should receive migraine-specific preventive therapy first. Established preventives remain valuable, particularly when comorbidities align. However, forced sequential failure through non-specific therapies should no longer be treated as a neutral administrative rule. In a patient with substantial disability, contraindications, prior intolerance, or high risk of progression, delayed access to appropriate prevention may itself become part of the disease pathway.

The practical change is small but consequential: migraine care should move from a visit-based model to a pathway-based model, consistent with headache service recommendations that emphasize structured care pathways, appropriate triage, and timely escalation, and with consensus guidance on integrating modern migraine treatments into clinical practice [5]. The visit-based model asks, What can we prescribe today? The pathway-based model asks, Where is this patient on the trajectory from episodic attacks to chronic disability, and what must happen next to interrupt that trajectory? Table 1 summarizes how this proposed precision-triage approach reframes common migraine-care questions into more actionable clinical decisions.

**Table 1:** Proposed precision triage pathway for migraine care, informed by headache service pathway recommendations and modern migraine treatment consensus guidance. Reference: [5]

Conventional care question	Precision-triage question	Clinical consequence
How many headache days?	How much disability, risk, and medication pressure?	Burden is not underestimated when frequency is moderate.
Has the patient failed older preventives?	Which preventive fits this patient now?	Treatment sequence is justified clinically, not inherited administratively.
Is the patient overusing medication?	Why has acute medication become the main survival strategy?	Medication overuse triggers support rather than blame.
Does the patient need a specialist?	What level of care is needed next, and how soon?	Referral becomes timely and purposeful.
Can digital tools collect data?	Can technology detect deterioration and improve accountability?	Digital health supports care rather than replacing it.

Artificial intelligence (AI) may help, but only if its role remains modest and accountable. AI could summarize headache diaries, flag rising acute medication days, identify missing red-flag documentation, prepare visit summaries, or translate treatment plans into patient-friendly language. Recent discussions in headache medicine recognize the relevance of such tools while emphasizing their risks [5]. AI should therefore be evaluated not only for accuracy, but for equity. A tool that accelerates care for digitally fluent, insured, urban patients while leaving others behind is not precision medicine; it is selective acceleration.

Equity is the real stress test of precision triage. Similar burden should produce similar attention regardless of geography, income, language, gender, digital literacy, or administrative persistence. The most important migraine pathway is not the one that works in an expert headache center for the already-referred patient. It is the one that helps primary care clinicians, emergency clinicians, and general neurologists recognize when a patient is drifting toward chronic disability and when prevention should no longer wait.

Conclusion

The next breakthrough in migraine medicine may be organizational rather than molecular. We already have diagnostic criteria, burden data, migraine-specific preventive options, and emerging digital tools. What remains unreliable is the bridge between these resources and the patient whose life is being narrowed by migraine. Precision triage offers a way to build that bridge: classify accurately, measure burden honestly, identify risk early, escalate rationally, and use technology only when it increases accountability. Migraine does not need to be made more dramatic to deserve attention. It needs to be made harder for systems to underestimate. If delayed access can worsen the migraine trajectory, then access is not a peripheral administrative issue. It is part of prevention.
